# Solution Structure of Tensin2 SH2 Domain and Its Phosphotyrosine-Independent Interaction with DLC-1

**DOI:** 10.1371/journal.pone.0021965

**Published:** 2011-07-12

**Authors:** Kun Dai, Shanhui Liao, Jiahai Zhang, Xuecheng Zhang, Xiaoming Tu

**Affiliations:** 1 Hefei National Laboratory for Physical Sciences at Microscale, School of Life Sciences, University of Science and Technology of China, Hefei, Anhui, People's Republic of China; 2 School of Life Sciences, Anhui University, Hefei, Anhui, People's Republic of China; University of Hong Kong, Hong Kong

## Abstract

**Background:**

Src homology 2 (SH2) domain is a conserved module involved in various biological processes. Tensin family member was reported to be involved in tumor suppression by interacting with DLC-1 (deleted-in-liver-cancer-1) via its SH2 domain. We explore here the important questions that what the structure of tensin2 SH2 domain is, and how it binds to DLC-1, which might reveal a novel binding mode.

**Principal Findings:**

Tensin2 SH2 domain adopts a conserved SH2 fold that mainly consists of five β-strands flanked by two α-helices. Most SH2 domains recognize phosphorylated ligands specifically. However, tensin2 SH2 domain was identified to interact with nonphosphorylated ligand (DLC-1) as well as phosphorylated ligand.

**Conclusions:**

We determined the solution structure of tensin2 SH2 domain using NMR spectroscopy, and revealed the interactions between tensin2 SH2 domain and its ligands in a phosphotyrosine-independent manner.

## Introduction

Src homology 2 (SH2) domain was firstly identified from N-terminal noncatalytic region of fujinami sarcoma virus P130^gag-fps^. It is a conserved domain of approximately 100 residues and shared by a number of cytoplasmic tyrosine kinase [1]. Few years after its identification, the SH2 domain was found in various proteins other than tyrosine kinases, such as PLCγ1, RasGAP, Grb2, suggesting its role as an interaction module [2]. Meanwhile, the works on GAP and EGF receptor verified the interactions between the SH2 domain and phosphorylated tyrosine (pTyr) containing protein [3]–[5].

Post-translational modification by phosphate group is a highly dynamic process, which is critical for regulation of various cellular events. Especially, phosphorylation/dephosphorylation of tyrosine residues of intracellular proteins is a critical signal for multiple biological events, e.g. DNA replication and repair, chromosome recombination and segregation, motility, metabolism, and gene transcription [6]. The diverse functions of SH2 domain containing proteins require broad but specific recognition of pTyr-ligands. Structural analyses of different SH2 domains revealed some common features of their structures and the binding between the SH2 domains and their ligands: (1) The SH2 domain mainly contains five anti-parallel β-strands flanked by two α-helices [7]. (2) The SH2 domain has two binding regions: one is the pTyr binding site, a deep positive-charged pocket [8], the other is the so-called “specificity determine region”, which includes residues close to the pTyr binding site [7]–[10]. It is revealed that the C-terminal three residues adjacent to pTyr are critical for the affinity and specificity of SH2 domain-ligand interaction [9], [10]. These provide SH2 domain containing proteins with properties of precise recognition of ligands and efficient regulation of signaling pathway. On the other hand, the SH2 domains binding pTyr through variable C-termini may facilitate SH2 containing proteins interacting with various ligands [7]. In most cases, SH2 domain-ligand binding is dependent on phosphorylated tyrosine.

Tensin is a family of proteins localized to focal adhesions of cells [11]. Tensin family has four members: tensin1, tensin2, tensin3 and cten. All these four tensins are proteins with multidomain, which allow tensins to interact with several ligands simultaneously. This property of tensin family reflects its essential role in signal transduction pathway. Although the sequence similarity between tensin members is low, they share some common features at N-terminus and C-terminus. For instance, all tensins have a SH2 domain and a phosphotyrosine binding domain (PTB domain) at C-terminus. In addition, tensin1, tensin2, tensin3 all possess actin binding domain (ABD) and focal adhesion binding site (FAB) at N-terminus [11]. Previous studies suggested that tensin2 interacts with DLC1 through PTB domain as well as SH2 domain [12]. Although PTB domain of tensin2 has been reported to play a more dominant role than tensin2 SH2 domain in recognizing DLC1 [12], SH2 domain in other tensin members seems to be preferential in DLC-1 recognition [13].

A previous work has revealed a relationship between tensin3 and tumorigenesis by showing a role of tensin3 in maintaining the transformed properties of cell lines from advanced cancers [14]. More recently, cten was considered to be a potential tumor suppressor by interacting with DLC-1 (deleted-in-liver-cancer-1), a member of RhoGTPase activating protein (GAP) family known to have suppressive activities in tumorigenicity and cancer metastasis [12], [13], [15]–[18]. This interaction was mediated by the SH2 domain of cten and the fragment “CSRLSIY^442^DNVPG” of DLC-1 [13]. Further experiments revealed that the interactions between cten SH2 domain and DLC-1 are phosphortyrosine independent [13]. This phosphorylation independent manner has also been observed for the SH2 domain-ligand binding of SAP, Grb7, and Vav1 [7], [13], [18]–[20], which might indicate a new binding mode of the SH2 domain.

In this study, we identified the interactions between the SH2 domain of tensin2, one member of tensin family, and the fragment “CSRLSIY^442^DNVPG” of DLC-1. Similar to cten, the interactions between the SH2 domain of tensin2 and DLC-1 were phosphortyrosine independent. The solution structure of tensin2 SH2 domain was determined by Nuclear Magnetic Resonance (NMR) spectroscopy. Tensin2 SH2 domain adopted a typical SH2 fold. Furthermore, chemical shift perturbation indicated a similar manner of tensin2 SH2 domain in binding to nonphosphorylated and phosphorylated ligands.

## Materials and Methods

### Sequence analysis

Sequence alignments were performed using ClustalW2 [21]. Further processing of the alignment files was carried out using ESPript 2.2 [22]. The existence of the SH2 domain in tensin2 was determined using SMART (http://smart.embl-heidelberg.de/). Accession number of the proteins used for alignments are listed as follows: Swiss-Prot, O60880, *Homo sapiens* SH2_SAP; Swiss-Port, P16277, *Mus musculus* SH2_BIK; Swiss-Port, Q56XZ1, *Arabidopsis thaliana* SH2_SHB; Swiss-Prot, P23615.1, *Saccharomyces cerevisiae* SH2_SPT6; Swiss-Prot, Q63HR2.2, *Homo sapiens* SH2_tensin2; Swiss-Prot, Q9HBL0, *Homo sapiens* SH2_tensin1; Swisst-Prot, Q68CZ2, *Homo sapiens* SH2_tensin3; and Swiss-Prot, Q8IZW8, *Homo sapiens* SH2_cten.

### Protein expression and purification

Sequence encoding the SH2 domain of human tensin2 was amplified and cloned into pET-22b (Novagen). Following primers were used for polymerase chain reaction (PCR): forward primer 5′-CCGCATATGGATACATCCAAGTTCTGGTAC-3′ and reverse primer 5′-TTCTCGAGTTTGCTGGGAATGCGCAGGCAGCAfoG-3′ (restriction endonuclease sites underlined). The recombinant tensin2 SH2 domain contained a C-terminal tag of six histidine residues. The recombinant protein was expressed in *Escherichia coli* strain BL21 Gold (DE3) and purified according to previous procedure [23]. ^15^N-labeled and ^13^C, ^15^N-labeled tensin2 SH2 domain were prepared in the same way, except that super broth was replaced by M9 medium containing 0.5 g/L ^15^N-labeled ammonium chloride and 2.5 g/L ^13^C-labeled glucose as the sole nitrogen source and carbon source, respectively. The purified protein was dialyzed with buffer containing 20 mM NaH_2_PO_4_, 150 mM NaCl, 50 mM arginine, 50 mM glutamine and 2 mM EDTA, pH 6.8, and concentrated to 0.6 mM for further NMR experiments.

### Site-directed mutagenesis

The site-directed mutagenesis (Y41S) was performed by PCR with the previousely constructed plasmid containing gene of Tensin2 SH2. The following primer sets were used: forward primer, 5′- CATTCATTCCAAGGAGCTTCTGGGCTGGCCCTCAAG -3′, reverse primer, 5′- GCCACCTTGAGGGCCAGCCCAGAAGCTCCTTGGAATG -3′. The PCR products were digested with DpnI (TaKaRa) for 10 hours, and then transformed into *E. coli* BL21. The plasmids containing mutated gene were sequenced to confirm the Y41S mutagenesis. The tensin2 SH2 (Y41S) were prepared as described above for further study.

### Peptide synthesis

Peptides “CSRLSIY^442^DNVPG” and “CSRLSI pY^442^DNVPG” (residues 436–447 of DLC-1 with Y^442^ nonphosphorylated/phosphorylated) were synthesized by GL Biochem (shanghai). The peptides were purified by HPLC (purity of peptides >95%), and molecular weight of peptides were confirmed by MS analysis.

### Surface plasmon resonance (SPR)

Real-time interactions between tyrosine-phosphorylated or nonphosphorylated peptide and tensin2 SH2 domain or mutated tensin2 SH2 domain (Y41S) were measured by surface plasmon resonance (SPR) on a Biacore 3000 (Biacore Inc.). SPR experiments were performed on sensor chips CM5 (carboxymethylated dextran, BiacoreAB). Chip was treated as described previously [23]. Purified tensin2 SH2 domain and mutated tensin2 SH2 domain were diluted with running buffer (20 mM NaH_2_PO_4_, 150 mM NaCl, 50 mM arginine, 50 mM glutamine and 2 mM EDTA, pH 6.8). The kinetic analysis of the interactions between phosphorylated peptide and tensin2 SH2 domain was performed at 6-step concentration of tensin2 SH2 domain (0.18 µM, 0.38 µM, 0.75 µM, 1.50 µM, 3.00 µM, and 6.00 µM) at a flow rate of 30 µL/min for 2 min, whereas the kinetic analysis of the interactions between nonphosphorylated peptide and tensin2 SH2 domain was performed at 6-step concentration of tensin2 SH2 domain (0.41 µM, 0.81 µM, 1.63 µM, 3.25 µM, 6.50 µM, and 13.00 µM). The kinetic analysis of the interactions between phosphorylated peptide and mutated tensin2 SH2 (Y41S) domain was performed at 6-step concentration of mutated tensin2 SH2 (Y41S) domain (0.50 µM, 1.00 µM, 2.00 µM, 4.00 µM, 8.00 µM, and 16.00 µM) at a flow rate of 30 µL/min for 2 min, whereas the kinetic analysis of the interactions between nonphosphorylated peptide and mutated tensin2 SH2 domain (Y41S) was performed at 6-step concentration of mutated tensin2 SH2 domain (Y41S) (3.13 µM, 6.25 µM, 12.50 µM, 25.00 µM, 50.00 µM, and 100.00 µM). All analyses were performed three times. To overcome refractive index changes, data of control surface were subtracted from that of peptide-immobilized surface. Interactions between peptide and Albumin Bovine V (BSA, Sigma, 8 mg/mL) were measured as a negative control. Kinetic analyses of SPR data were performed using BIAevaluation 4.1 (Biacore Inc.). Curves were fitted with 1∶1 (langmuir) binding model. The equilibrium dissociation constant (KD) was derived from kinetic analysis.

### Circular dichroism (CD) spectroscopy

CD experiments were performed on a Jasco-810 spectrophotometer (JASCO, Japan) over the wavelength range from 200 to 260 nm at 20°C. Tensin2 SH2 domain was dissolved in buffer (20 mM NaH_2_PO_4_, 150 mM NaCl, 50 mM arginine, 50 mM glutamine and 2 mM EDTA, pH 6.8) to a concentration of ∼0.6 mg/mL. Different molar ratios of SH2/peptide (1∶1, 1∶2 and 1∶3) were prepared in the same buffer. Measurements were taken in a 1 mm path-length quartz cuvette at a rate of 50 nm/min and data pitch of 1 nm. CD spectra of the peptides at corresponding concentrations were measured as control and subtracted. Three successive scans were recorded and averaged. The analyses of CD spectra were performed using Jascow32 software (JASCO, Japan).

### NMR spectroscopy, data processing and structure calculation

NMR samples were dissolved in buffer containing 20 mM NaH_2_PO_4_, 150 mM NaCl, 50 mM arginine, 50 mM glutamine, 2 mM EDTA, 90% H_2_O/10% D_2_O and pH 6.8. All NMR measurements were performed at 293 K on a Bruker DMX500 spectrometer. The following spectra were recorded to obtain backbone and side chain resonance assignments: ^1^H-^15^N HSQC, HNCO, HN(CA)CO, CBCA(CO)NH, CBCANH, H(CC)ONH, HBHA(CO)NH, HC(CO)NH. 3D ^15^N-edited and ^13^C-edited NOESY were acquired with mixing times of 100 and 130 ms, respectively. To identify the slowly exchanging amides, a ^15^N-labeled sample was lyophilized and dissolved in 99.96% D_2_O. Immediately, a series of ^1^H-^15^N HSQC were recorded to monitor the attenuation of NH signals. NMR data were processed using NMRPipe and NMRDraw [24], and then analyzed using Sparky 3 [25]. All software was run on a Linux system.

3D ^15^N-edited and ^13^C-edited NOESY spectra were used to determine the NMR distance restraints for structure calculations. Backbone torsion angle restraints were predicted from chemical shifts of five types of nuclei: ^13^Cα, ^13^Cβ, ^13^CO, ^1^Hα, and ^15^NH by using TALOS+ [26]. Hydrogen bond restraints were obtained by identifying slow-exchange amide protons after 12 hours' incubation following solvent exchange. For hydrogen bonds, two distance restraints were used: 2.0 Å for H-O and 3.0 Å for N-O. Structures were calculated using the program CYANA 3.0 [27]. A total of 200 conformers were independently calculated, and 20 lowest-energy structures were selected and analyzed by MOLMOL [28] and PROCHECK [29] online (http://nihserver.mbi.ucla.edu/SAVES_3/).

### Chemical shift perturbation

To identify the residues responsible for binding to tyrosine-phosphorylated or nonphosphorylated DLC-1 peptide in tensin2 SH2 domain, 0.5 mM ^15^N-labeled protein were titrated with unlabeled tyrosine-phosphorylated or nonphosphorylated peptide to different molar ratios (1∶0, 1∶1, 1∶2 and 1∶3, SH2/peptide). ^1^H-^15^N HSQC spectrum was acquired for analysis. Peptide stock solutions in identical buffer were titrated stepwise with a sample dilution of less then 10%. Combined chemical shift perturfobation was calculated using the following equation: 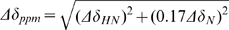. Δδ_NH_ and Δδ_N_ are the chemical shift variation in the proton and nitrogen dimensions, respectively. The threshold values used as a criterion for significance were 60% for intensity reduction and 0.1 ppm for chemical shift perturbation.

## Results

### Comparison of the primary sequence of the SH2 domain between tensins and other proteins

**Figure 1:**
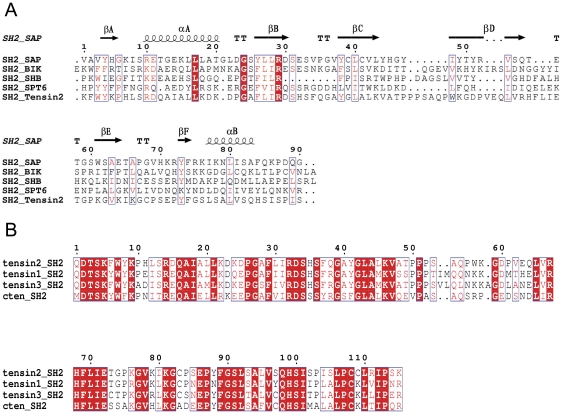
Multiple sequence alignments between different SH2 domains of proteins from different species. Alignments were performed using ClustalW2 and ESPript 2.2. Identical residues were shaded in red box. A. SH2 domains share low primary sequence similarity, but similar secondary structure. B. SH2 domains of tensin family members share high primary sequence similarity.

The SH2 domains have been found in hundreds of proteins involved in a variety of cellular processes. The SH2 domains from different proteins share low sequence identity (Figure 1A). For example, the SH2 domain of SAP which is related to SLAM-induced signal-transduction events in T lymphocytes shares only about 24% sequence identity with tensin2 SH2 domain. The sequence diversity of the SH2 domains may reflect their ability of binding various ligands in different cellular processes. Although the SH2 domains vary dramatically in sequence and ligand recognition, the SH2 domains from different tensin family members display 70%–80% of sequence identity between each other (Figure 1B), which may imply their common properties not only in structure but also in ligand recognition.

### Interactions between tensin2 SH2 domain and DLC-1 peptides

**Figure 2:**
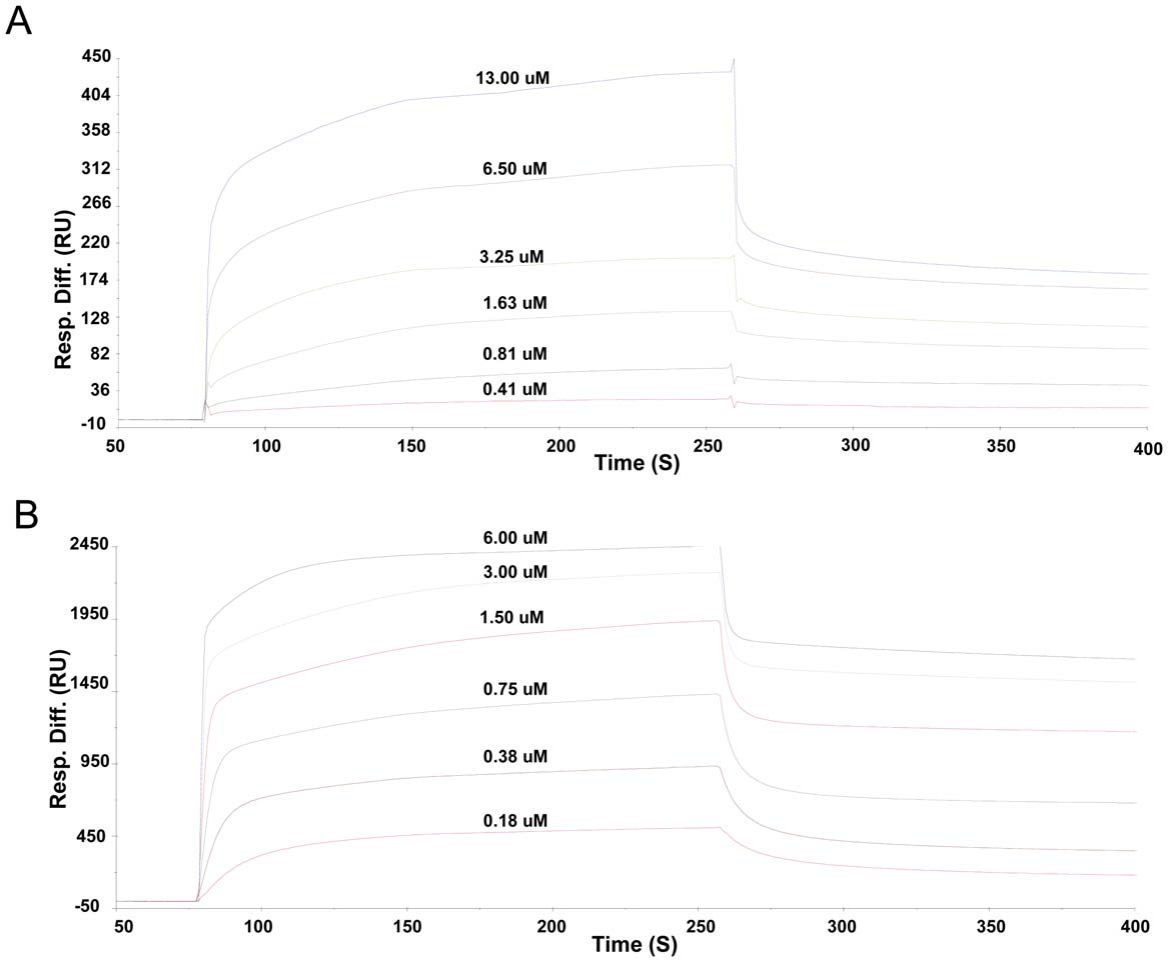
Kinetic analyses of interactions between tensin2 SH2 domain and nonphosphorylated/phosphorylated peptides by SPR. Kinetic analyses of interactions between peptides and tensin2 SH2 domain were performed at 6 steps of concentration of recombinant SH2 domain at a flow rate of 30 µL/min for 2 mins. A. SPR spectra of the SH2 domain binding to nonphosphorylated peptide. B. SPR spectra of the SH2 domain binding to phosphorylated peptide. Analyses were performed three times at each step of concentration. KD of the SH2 domain binding different peptide was derived from kinetic analysis.

Recently, the SH2 domain of tensin family protein cten that is able to bind DLC-1 peptides in a pTyr independent manner has been reported [12], [13]. To understand whether the SH2 domain of another tensin family member, tensin2, binds to the same ligand and whether this binding is also phosphorylation-independent, we investigated the interactions between tensin2 SH2 domain and non/phosphorylated DLC-1 peptides by SPR. The results indicated that tensin2 SH2 domain bound specifically to both phosphorylated and nonphosphorylated peptides. The equilibrium dissociation constant (KD) derived from kinetic analyses was about 248.00±8.82 nM for SH2-nonphosphorylated peptide interaction (Figure 2A) and 31.00±0.50 nM for SH2-phosphorylated peptide interaction (Figure 2B). It was surprising that phosphorylation of DLC-1 peptide increased its affinity to tensin2 SH2 domain by only eight folds approximately under the condition for SPR experiments, whereas for typical SH2 domain, the affinity of tyrosine-phosphorylated peptide to the SH2 domain is about four orders of magnitude greater than that of nonphosphorylated counterpart [7], [30]. This result indicated that the peptide recognition of tensin2 SH2 domain was relatively phosphorylation independent.

### Secondary structures of tensin2 SH2 domain in free form and in complex with DLC-1 peptides

**Figure 3:**
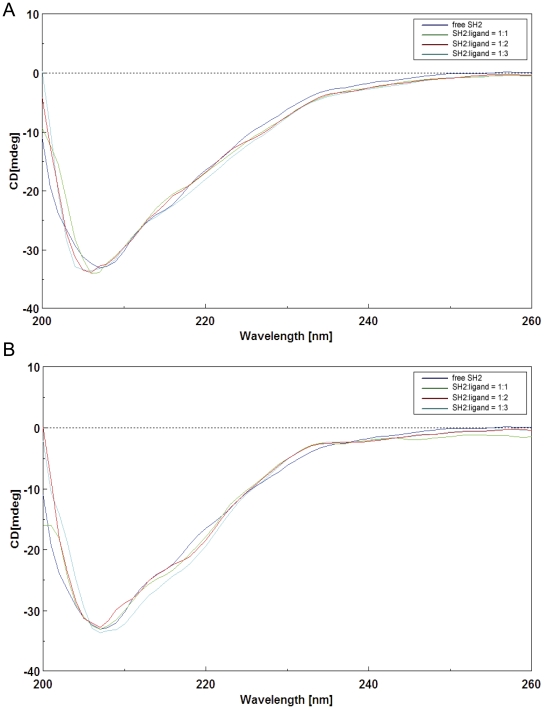
CD spectra of tensin2 SH2 domain free and in complex with peptides. A. CD spectra of tensin2 SH2 domain with nonphosphorylated peptide in different molar ratios (blue, green, red and cyan represent SH2/peptide ratios of 1∶0, 1∶1, 1∶2 and 1∶3, respectively). B. CD spectra of the SH2 domain with phosphorylated peptide in different molar ratios (blue, green, red and cyan represent SH2/peptide ratios of 1∶0, 1∶1, 1∶2 and 1∶3, respectively).

To investigate the secondary structure of tensin2 SH2 domain and its change upon binding to ligand, CD experiments were performed. The CD spectra of tensin2 SH2 domain in complex with non/phosphorylated DLC-1 peptides in different ratios indicated that there was no significant change in the secondary structure of tensin2 SH2 domain after binding to the ligands (Figure 3A and Figure 3B).

### Solution structure of tensin2 SH2 domain

**Figure 4:**
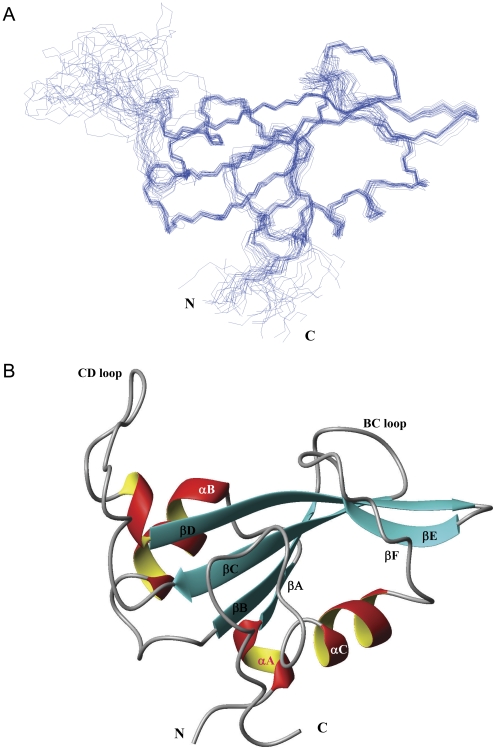
The NMR structure of tensin2 SH2 domain. A. Superposition of 20 lowest-energy NMR structures. B. The lowest-energy conformation used for ribbon representation on highlighting the six β-strands and two main α-helices that are identified in tensin2 SH2 domain.

Tensin2 SH2 domain, including residues from 1135 to 1248 of the protein, was recombinantly expressed and purified. The recombinant product exhibited good solubility and stability. The solution structure of tensin2 SH2 domain was calculated based on a series of NMR spectra. The NMR data used for structure calculations are summarized in Table 1. The chemical shift assignments of tensin2 SH2 domain have been deposited in the Biological Magnetic Resonance Data Bank (accession number 17314). The assembly of the 20 lowest-energy structures is shown in Figure 4A. Structures of tensin2 SH2 domain have been deposited in Protein Data Bank (PDB ID code 2l6k). The statistical parameters in Table 1 indicate a high-quality NMR structure of tensin2 SH2 domain. Calculated structure of tensin2 SH2 domain shows a characteristic SH2 fold, which includes the two main α-helices packing against either side of a central β sheet. The lowest-energy structure was shown in Figure 4B. The central β sheet is composed of three long antiparallel β strands (βB, βC, βD) followed by one short antiparallel β strand (βE) and one shorter β-sheet-like structure (βF). The two main α-helices (αB and αC) flank either side of the central β sheet. The residues composing secondary structures are listed as follows: residue 4 to 7 (αA), residue 8 to 9 (βA), residue 13 to 22 (αB), residue 29 to 33 (βB), residue 40 to 46 (βC), residue 66 to 72 (βD), residue 78 to 81 (βE), residue 87 to 89 (βF) and residue 91 to 98 (αC), from N-terminus to C-terminus.

**Table 1:** NMR structural statistics.

NMR restraints in the structure calculation	
Intraresidue	333
Sequential (|i - j| = 1)	447
Medium-range (|i - j| <5)	252
Long-range (|i - j| >/ = 5)	369
Hydrogen bonds	66
Total distance restraints	1580
Dihedral angle restraints	113
Residual violations	
CYANA target functions, Å	1.43±0.24
NOE upper distance constrain violation	
Maximum, Å	0.20±0.04
Number >0.2 Å	0±1
Dihedral angle constrain violations	
Maximum, °	3.23±0.72
Number >5°	0±0
Vander Waals violations	
Maximum, Å	0.30±0.00
Number >0.2 Å	3±1
Average structural rmsd to the mean coordinates, Å	
Secondary structure backbone^a^	0.31
Secondary structure heavy atoms^a^	0.80
All backbone atoms^b^	1.30
All heavy atoms^b^	1.79
Ramachandran statistics, %of all residues	
Most favored regions	81.5
Additional allowed regions	18.5
Generously allowed regions	0
Disallowed regions	0

a Includes residues in secondary structure.

b Obtained for residues T3-R111 since no long-range NOEs were identified for amino acid 1–2 and 112–115.

### Structural comparison with other SH2 domains

**Figure 5:**
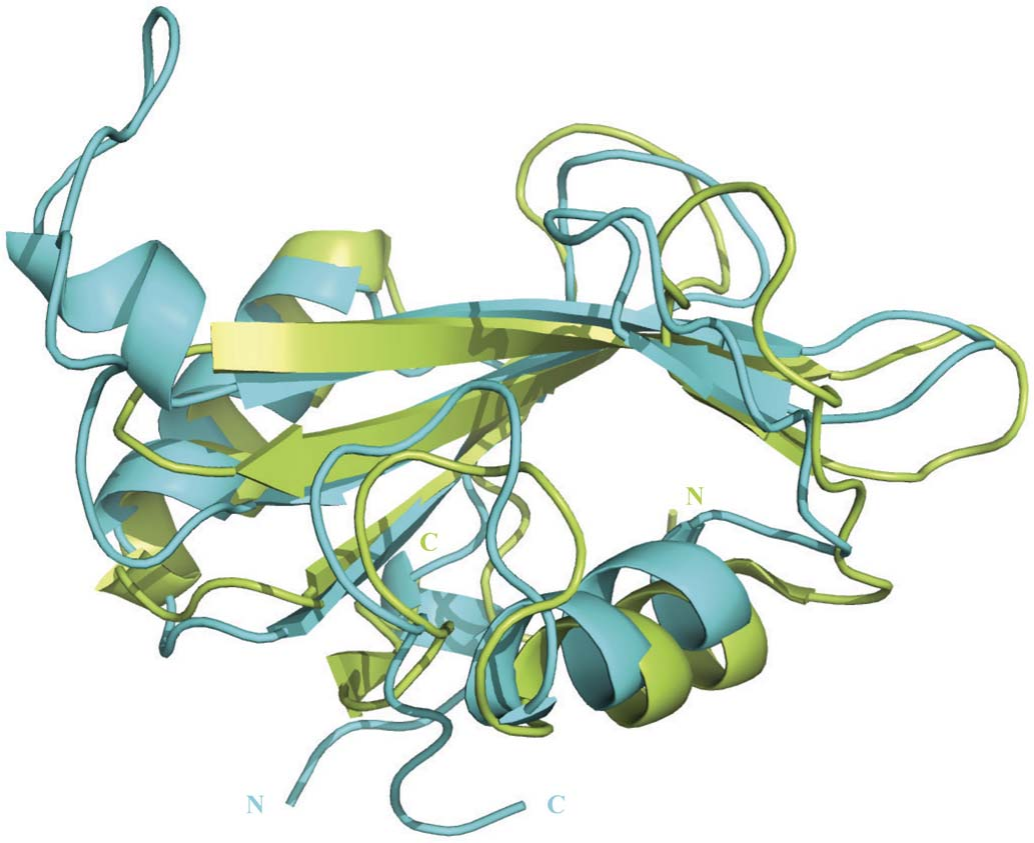
Structural comparison of tensin2 and SAP SH2 domains. The structure of SAP SH2 domain was shown in limon. The lowest-energy conformation of tensin2 SH2 domain was shown in cyan. The alignment was performed by using Pymol.

As the SH2 domains of tensin2 and SAP both interact with ligands in a pTyr-independent way, the structure of free SAP was used to perform structural compare. The alignment of SAP and the lowest-energy structure of tensin2 SH2 domain was shown in Figure 5. Although the two SH2 domains share low sequence identity, both structures show typical SH2 fold, with a central five β strands flanked by two α helices at either side, which demonstrated that the SH2 domain is a structurally conserved. A DALI [31] search using tensin2 SH2 domain as query sequence indicates that SH2 domains share high similarity in 3D-structure. The RMSD and Z-score between tensin2 SH2 domain and SAP are 2.4 Å and 10.8, respectively. The structural difference between these two SH2 domains lies in the loop between βC and βD. Tensin2 SH2 domain has a longer loop than SAP, implying that the former structure might be more flexible than that of the latter in this region.

### Identification of the residues of tensin2 SH2 domain involved in binding to DLC-1 peptides

**Figure 6:**
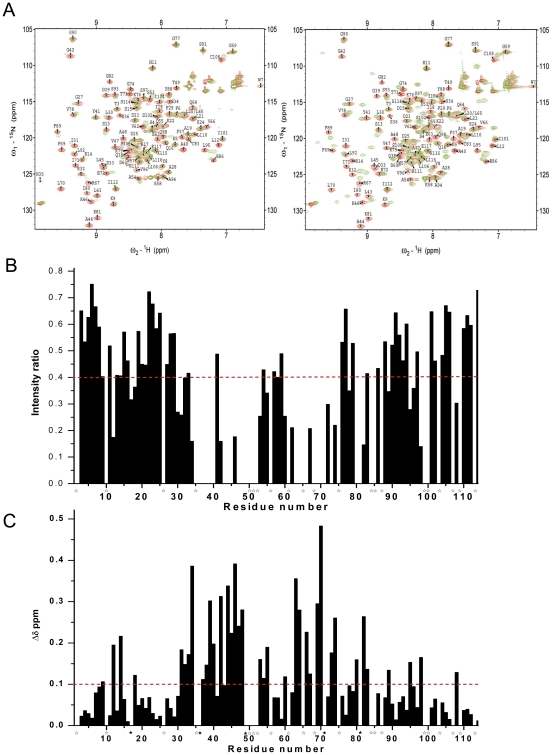
Identification of residues of tensin2 SH2 domain for binding to nonphosphorylated/phosphorylated ligands. A. The ^1^H-^15^N HSQC spectra of the ^15^N-labeled tensin2 SH2 domain free (red) and titrated with its ligand (left: nonphosphorylated peptide; right: phosphorylated peptide) (green) were overlaid. B. The ratio of peak intensity after compared with before the addition of nonphosphorylated peptide. C. The chemical shift changes after the addition of phosphorylated peptide. All the comparison was performed at the SH2/peptide molar ratio of 1∶3. The broken lines represent the significance level of intensity reductions >60% (intensity ratios <40%) and chemical shift changes >0.1 ppm, respectively. The residues that had significant changes and were untraceable in ^1^H-^15^N HSQC spectra after the addition of phosphorylated peptide were labeled with ★; the residues that could not be assigned were labeled with ⋆.

To determine the residues in tensin2 SH2 domain involved in recognition of non/phosphorylated DLC-1 peptides, chemical shift perturbation experiments were performed. ^1^H-^15^N HSQC experiments were recorded for ^15^N-labeled tensin2 SH2 domain before and after addition unlabeled nonphosphorylated or phosphorylated peptides to different molar ratios of SH2/peptide. The spectral changes that occurred after peptide addition were characterized by resonance intensity reductions and chemical shift changes. The residues of tensin2 SH2 domain interacting with nonphosphorylated ligand were signified by the obviously reduced peak intensity. Compared with it, the residues of tensin2 SH2 domain interacting with phosphorylated ligand were signified by the substantial resonance shift (Figure 6A to 6C). The amino acids affected in the presence of the nonphosphorylated ligand were residues Leu12, Ala17, Ile18 (belong to αB), Leu30-Arg32 (belong to βB), Ser34, Ser36-Gly39 (belong to BC loop), Ala40, Gly42-Lys46 (belong to βC), Val47-Thr49, Ser53, Gln55, Asp60, Val62-Gln64 (belong to CD loop), Val66, Arg67, Phe69-Glu72 (belong to βD), Thr73, Gly74 (belong to DE loop), Val78, Ile80, Lys81 (belong to βE), Gly82 (belongs to EF loop), Phe89 (belongs to βF), Leu95, Val96, Gln98 (belong to αC) and Cys108 (belongs to C-terminal coil) (Figure 6B). The amino acids affected in the presence of phosphorylated ligand were residues Lys9 (belongs to βA), Leu12, Arg14, Ala17, Ile18 (belong to αB), Ile31-Asp33 (belong to βB), Ser34, Ser36-Gly39 (belong to BC loop), Ala40, Gly42-Lys46 (belong to βC), Val47-Thr49, Ser53-Gln55, Asp60, Glu63, Gln64 (belong to CD loop), Val66, Arg67, Phe69-Ile71 (belong to βD), Thr73, Gly74 (belong to DE loop), Ile80, Lys81 (belong to βE), Gly82, Cys83 (belongs to EF loop), Phe89 (belongs to βF), Leu95, Gln98 (belong to αC) and Cys108 (belongs to C-terminal coil) (Figure 6C). These residues might be involved in the interactions between tensin2 SH2 domain and the ligands. The distribution of the residues which showed intensity reductions in the presence of nonphosphorylated ligand was almost identical to that of residues which showed chemical shift perturbations in the presence of phosphorylated ligand, which might reflect the similar binding pattern of tensin2 SH2 domain to peptides with different phosphorylation status.

Tyr41 of tensin2 SH2 domain was shown to be affected both in the presence of phosphorylated and nonphosphorylated ligands in chemical shift perturbation experiments. Meanwhile, it has been reported previously that tensin3 SH2 domain's Tyr1206, which was the counterpart of Tyr41 in tensin2 SH2 domain, is important for ligand binding [14]. Therefore, we mutated the Tyr41 residue to Ser residue and tested how this mutation affected their interactions. The equilibrium dissociation constant (KD) derived from SPR experiment was about 16.00±0.20 µM for the Y41S mutant-nonphosphorylated peptide interactions (Figure S1A) and 5.00±0.04 µM for the Y41S mutant-phosphorylated peptide interactions (Figure S1B). The KD value of Y41S mutant-ligands reduced by two orders compared with that of tensin2 SH2 domain-ligands, which suggested an important role of Tyr41 of tensin2 SH2 domain both in the recognition of the nonphosphorylated and phosphorylated ligands.

**Figure 7:**
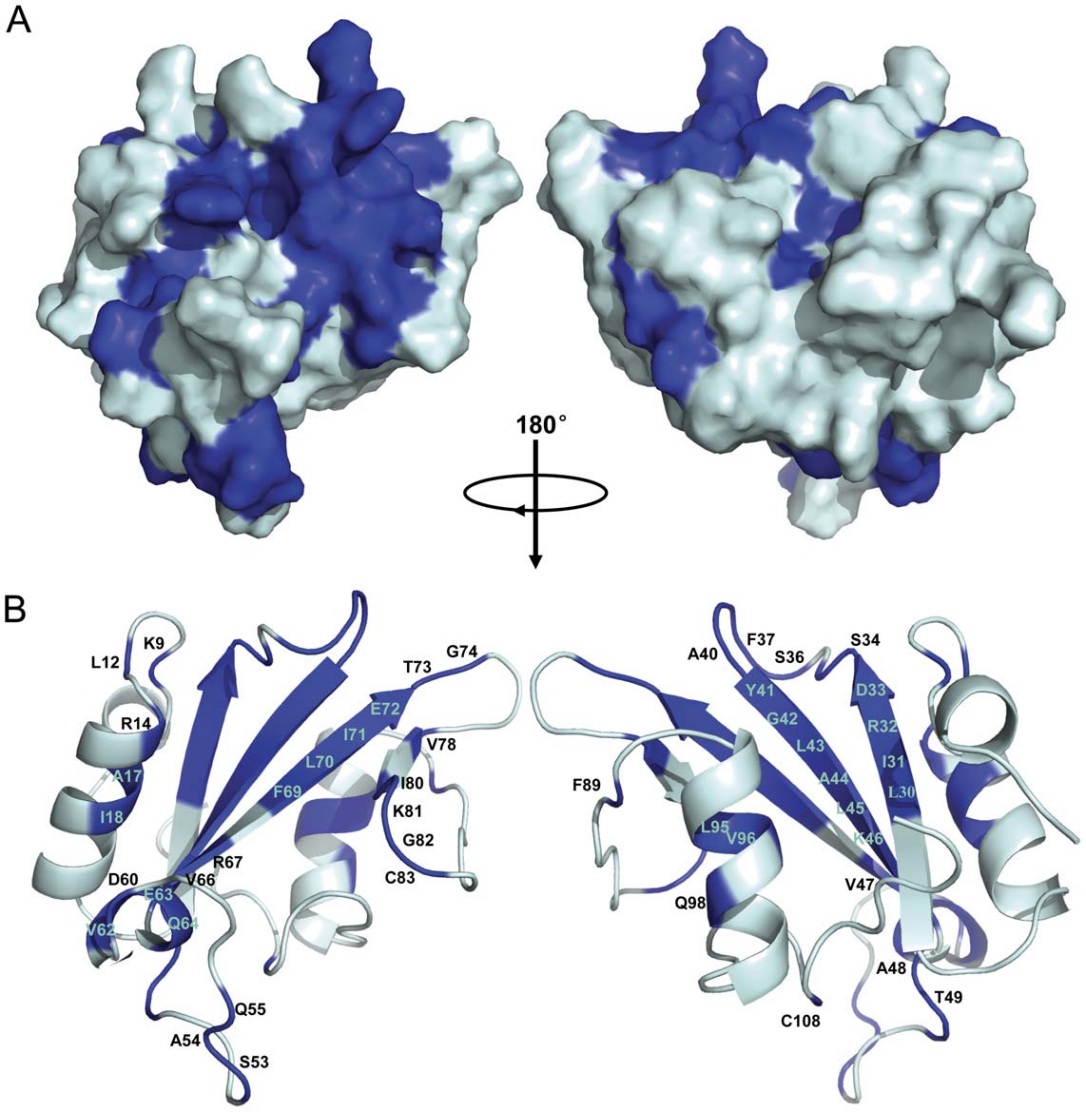
Molecular surface and ribbon representation of tensin2 SH2 domain displaying residues involved in its interaction with nonphosphorylated/phosphorylated ligand. A. Molecular surface of tensin2 SH2 domain. B. Ribbon representation of tensin2 SH2 domain. Molecular surface and ribbon representation of tensin2 SH2 domain were rotated by 180° horizontally. Residues marked in blue corresponded to the ones involved in the interactions with both nonphosphorylated and phosphorylated ligands.

Based on chemical shift perturbation, the structure of tensin2 SH2 domain was shown with residues marked to display the ones involved in peptide interaction (Figure 7). It is remarkable that the majority of significantly affected residues, such as Leu30, Ile31, Ser36 to Ala40, Gly42 to Thr49, Val62 to Gln64, Arg69 to Gly74 whose intensity reductions were higher than 70% even to 100% and Ser34, Gly39, Ala44, Lys46, Glu63 whose chemical shift perturbations were bigger than 0.3 ppm, were located in the central anti-parallel β-sheet and the loops between β strands. These sites are the conserved binding sites for typical SH2 domains recognizing their ligands. In addition, residues Lys9 (belongs to βA), Leu12, Arg14, Ala17 and Ile18 (belong to αB), which are located outside the central region, displayed discernable changes during titration as well. These residues may also participate in the interactions between tensin2 SH2 domain and its ligands.

## Discussion

Tensin is a family containing four members that are localized to the cytoplasmic side of focal adhesions and play essential roles in extracellular matrix based signaling pathway. Previous studies have suggested cten, a tensin family member, may function as a tumor suppressor, through the interaction with DLC-1 [13] that has been found to be absent or suppressed in many cancers such as hepatocellular carcinoma (HCC). However, more recent studies revealed other roles of cten as an oncogene in colorectal cancer through enhancing the colony formation, anchorage-independent growth, cell migration and invasion [32], [33]. Mutations in DLC-1 which disrupt its interaction with cten SH2 domain will abolish its role as a tumor suppressor [34]. Thus, cten is important for the functions of DLC-1 in tumor suppression. In addition, tensin3 has also been reported to be involved in tumorigenesis [14]. In this study, we indicate that another member of tensin family, tensin2, is able to interact through its SH2 domain with DLC-1 as well; and the interaction is pTyr-independent, similar to cten SH2 domain. The interaction of tensin2 SH2 domain with tumor suppressor DLC-1 might reflect the general roles of tensin family members in preventing tumor development.

So far, no structure has been reported for the SH2 domains of tensin family proteins. Though the sequence similarity between tensin members is low, their SH2 domains share high sequence identity, which implies they might possess conserved structure. In this study, we reported the NMR solution structure of tensin2 SH2 domain. The structure is mainly composed of five β-strands flanked by two α-helices at either side, which is a characteristic of typical SH2 domain, suggesting that SH2 domain family is highly conserved in structure.

Although tensin2 SH2 domain adopts a similar structure to other SH2 domains, it recognizes its ligand in a pTyr-independent instead of pTyr-dependent manner. SAP, which has only one SH2 domain, was the first protein reported to interact with ligand in a pTyr-independent manner. The interaction mechanism has been disclosed by analyzing its free structure and complex structures with phosphorylated/nonphosphorylated ligands. A theory called “three-pronged plug and socket” was proposed to illustrate the pTyr-independent interaction. This theory suggests three distinct regions on SAP mediate specific interaction with N-terminus, residue Tyr, and C-terminus of ligand, respectively [35]. Ligands of SAP share a motif of “T/S-I-Y-X-X-V/I” (X represents any residue) [35]. Although the SH2 domain of tensin2 shares only about 24% of the primary sequence identity with that of SAP, the ligand of tensin2 SH2 domain, DLC-1, contains the motif of “T/S-I-Y-X-X-V/I” as well, within the sequence of “CSRLSIYDNVPG”. The same motif recognized by SAP and tensin2 SH2 domains suggests that they may interact with ligands in a similar way.

Classical SH2 domains bind to phosphorylated peptides through a conventional phosphotyrosine-binding pocket. In our study, chemical shift perturbation and structural comparison indicate that tensin2 SH2 domain maintains this general binding feature. The two conserved binding regions existing in tensin2 SH2 domain are as follows: (1) Residues binding to DLC-1 ligands regardless of the phosphorylation status are located in the central anti-parallel β-sheets and the loops between βB and βC, including Leu30, Ile31, Arg32, Asp33, Ser34, Ser36, Phe37, Gln38, Gly39, Ala40, Tyr41, Gly42, Leu43, Ala44, Leu45 and Lys46. These residues might form the pTyr or Tyr binding site. Notably, Arg32, in βB strand of tensin2 SH2 domain, which is also present in other SH2 domains, is essential for recognition of the Tyr residue of ligand. (2) Residues (including Ile80, Lys81, Gly82, Phe89 and Cys108) located in βE, βF, the loop between βE and βF, and C-terminal coiled coil that correspond to so-called “specificity determine region” might be responsible for the interactions between tensin2 SH2 domain and C-terminus of ligand. Additionally, the third group of residues, located in βA and αB, including Lys9, Leu12, Arg14, Ala17 and Ile18, were identified. Similar as those of SAP SH2 domain, these residues may participate in recognizing the N-terminus of ligand and be required for its specific phosphorylation-independent binding.

In summary, we determined the solution structure of tensin2 SH2 domain and identified the pTyr-independent interactions between tensin2 SH2 domain and DLC-1 fragment. Our data imply the SH2 domains of tensin family might have conserved structure, common mechanism of ligand recognition in a pTyr-independent way.

## Supporting Information

Figure S1 **Kinetic analyses of interactions between mutated tensin2 SH2 domain and nonphosphorylated/phosphorylated peptides by SPR.** Kinetic analyses of interactions between peptides and mutated tensin2 SH2 domain were performed at 6 steps of concentration of recombinant SH2 domain at a flow rate of 30 µL/min for 2 mins. A. SPR spectra of the mutated SH2 domain (Y41S) binding to nonphosphorylated peptide. B. SPR spectra of the mutated SH2 domain (Y41S) binding to phosphorylated peptide. Analyses were performed three times at each step of concentration. KD of the SH2 domain binding different peptide was derived from kinetic analysis.
(TIF)
