# Supplementary figures and images for: Solution Structure of Tensin2 SH2 Domain and Its Phosphotyrosine-Independent Interaction with DLC-1
Source: PLoS One. 2011 Jul 12;6(7):e21965. doi: 10.1371/journal.pone.0021965 (PMC3134462; doi:10.1371/journal.pone.0021965)

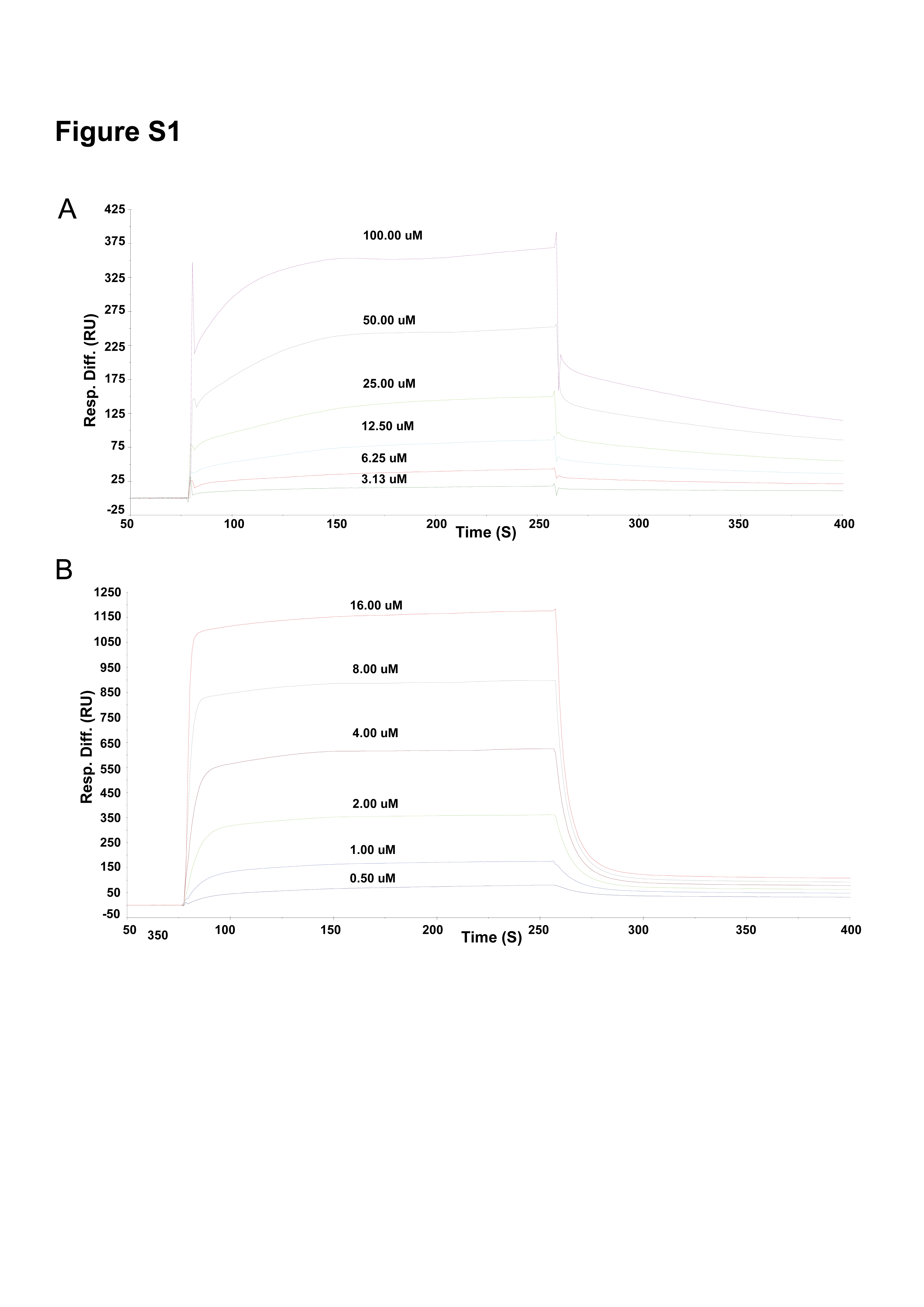

Supplement: Figure S1 — Kinetic analyses of interactions between mutated tensin2 SH2 domain and nonphosphorylated/phosphorylated peptides by SPR. Kinetic analyses of interactions between peptides and mutated tensin2 SH2 domain were performed at 6 steps of concentration of recombinant SH2 domain at a flow rate of 30 µL/min for 2 mins. A. SPR spectra of the mutated SH2 domain (Y41S) binding to nonphosphorylated peptide. B. SPR spectra of the mutated SH2 domain (Y41S) binding to phosphorylated peptide. Analyses were performed three times at each step of concentration. KD of the SH2 domain binding different peptide was derived from kinetic analysis. (TIF) [file pone.0021965.s001.tif]
